# Performance of Commercial Deep Learning-Based Radiation Dose Optimization Software in Pediatric Radiology: A Systematic Review

**DOI:** 10.3390/children13091147

**Published:** 2026-08-26

**Authors:** Curtise K. C. Ng

**Affiliations:** 1 Curtin School of Diagnostic and Therapeutic Sciences, Curtin University, GPO Box U1987, Perth, WA 6845, Australia; curtise.ng@curtin.edu.au or curtise_ng@yahoo.com.hk; Tel.: +61-8-9266-7314; 2 Curtin Medical Research Institute (Curtin MRI), Faculty of Health Sciences, Curtin University, GPO Box U1987, Perth, WA 6845, Australia

**Keywords:** artificial intelligence, as low as reasonably achievable, children, computed tomography, convolutional neural network, dose reduction, image processing, machine learning, medical imaging, noise

## Abstract

**Highlights:**

**What are the main findings?**

- Commercial computed tomography (CT) deep learning-based image reconstruction (DLIR) software achieved radiation dose reductions in pediatric radiology ranging from 11.2% to 97.9% while maintaining image quality, with some studies reporting image quality superior to the reference standard, suggesting further dose reduction potential.
- More than three-quarters of the included studies reported radiation dose reductions of at least 50% without compromising image quality in pediatric CT.

**What are the implications of the main findings?**

- Commercial CT DLIR software shows substantial potential for radiation dose optimization in pediatric CT and supports further prospective, multi-center clinical validation before broader implementation.
- Future studies should further evaluate commercial DLIR products, including applications in positron emission tomography and X-ray imaging, using diverse pediatric populations, adequately sized prospective datasets, effective dose assessment, and clinically relevant outcome measures such as diagnostic confidence/quality/accuracy.

**Abstract:**

**Background/Objectives**: To date, no systematic review has focused specifically on commercially available deep learning-based image reconstruction (DLIR) software for radiation dose optimization in pediatric radiology. The purpose of this article was to systematically review original clinical studies evaluating the performance of commercial DLIR software for radiation dose optimization in pediatric radiology and to assess their methodological quality. **Methods**: A literature search was conducted on 28 April 2026 using seven electronic databases. The review was registered with the Open Science Framework (Registration DOI: 10.17605/OSF.IO/3JDQS). **Results**: Thirteen papers met the selection criteria and were included in the review. These studies evaluated five commercial computed tomography (CT) DLIR software products. Clinically achievable radiation dose reductions ranged from 11.2% to 97.9% without compromising image quality, and in some cases, even exceeding that of the reference standard, suggesting further dose reduction potential. Excluding two studies that reported substantially lower dose reductions, the clinically achievable dose reduction range was 36.0–97.9%. Furthermore, more than three-quarters of the included articles reported dose reductions of at least 50% while maintaining image quality. Improvements in study methodology were evident among papers published from 2025 onward. **Conclusions**: Commercial CT DLIR software can achieve substantial radiation dose reductions in pediatric CT while maintaining image quality. However, further clinical studies are needed to evaluate a broader range of commercial DLIR software, including applications in positron emission tomography and X-ray imaging. Future studies should ideally include the full pediatric age range, prospectively collected and adequately sized datasets from underrepresented geographic regions, effective dose assessment, and clinically meaningful outcome measures such as diagnostic confidence/quality/accuracy.

## 1. Introduction

The use of artificial intelligence (AI), particularly deep learning (DL), has become increasingly prevalent in pediatric radiology [1,2,3,4,5]. One notable application is deep learning-based image reconstruction (DLIR), which has been developed for radiation dose optimization, i.e., reducing radiation dose while maintaining image quality [3,4,5]. This is especially important in pediatric populations, as children are more sensitive to ionizing radiation than adults and have a longer lifespan, allowing potential radiation-induced effects to manifest over time [4,5,6]. Although the radiation doses involved in most radiological examinations are generally considered low (less than 100 mSv), their long-term effects remain uncertain [4,5]. Furthermore, the principle of “as low as reasonably achievable” (ALARA) underpins radiological practice, emphasizing the need to minimize radiation exposure without compromising image quality to a level that adversely affects diagnostic accuracy [4,5,7].

DLIR for radiation dose optimization is primarily a form of medical image denoising. However, it extends conventional denoising approaches by reducing image noise while preserving spatial resolution [8,9,10,11]. Given the importance of radiation dose optimization in pediatric radiology, several commercial software products have become available for clinical use. These include AlgoMedica’s PixelShine (Sunnyvale, CA, USA) [12], Canon Medical Systems’ Advanced Intelligent Clear-IQ Engine (AiCE) (Tochigi, Japan) [13,14], ClariPI’s ClariCT.AI (Seoul, Republic of Korea) [15], General Electric (GE) Healthcare’s TrueFidelity (Chicago, IL, USA) [15,16,17,18], and Philips Healthcare’s Precise Image (Best, The Netherlands) for computed tomography (CT) [19]; Subtle Medical’s SubtlePET (Menlo Park, CA, USA) for positron emission tomography (PET) [20]; and Canon Medical Systems’ Intelligent Noise Reduction (INR) [21,22] and Samsung Electronics Co., Ltd.’s SimGrid (Suwon-si, Republic of Korea) for X-ray imaging [23].

Nevertheless, concerns have been raised in recent years regarding the performance of commercial DLIR systems in pediatric radiology by pediatric radiologists and professional bodies, including the American College of Radiology (ACR), Asian Oceanic Society of Paediatric Radiology (AOSPR), European Society of Paediatric Radiology (ESPR), Sociedad Latinoamericana de Radiologia Pediatrica (SLARP), Society of Paediatric Neuroimaging (SPIN), and Society for Paediatric Radiology (SPR) [24,25,26]. These concerns include the fact that many commercial DLIR products are not specifically indicated for pediatric use [26,27,28]. Furthermore, even when systems are indicated for pediatric use, the substantial variability in anatomy and pathology across pediatric age groups may lead to underrepresentation of certain subgroups in training datasets. This may result in suboptimal or unpredictable performance in specific clinical scenarios [26,27]. Consequently, these organizations emphasize the need for robust clinical efficacy evidence to support the use of DLIR systems in pediatric populations [26].

In recent years, only one narrative review and one systematic review have been published on DLIR in pediatric radiology (2021 and 2022, respectively) [4,5]. The narrative review focused on the basic principles, image characteristics, and application areas of DLIR in CT [5]. The existing systematic review evaluated the performance of DLIR systems across various pediatric radiology applications, including both in-house and commercial models [4]. However, its findings may have limited clinical applicability due to the inclusion of in-house models, which are not widely accessible and present challenges in implementation [4,29,30]. In the absence of a systematic review focused specifically on commercially available DLIR software, it remains difficult for clinicians to determine whether implementation in routine practice is justified. This lack of high-quality, synthesized evidence may hinder broader clinical adoption and limit the potential benefits of DLIR in achieving radiation dose optimization and adherence to the ALARA principle in pediatric radiology [29,30]. The purpose of this article was to systematically review original clinical studies to evaluate the performance of commercial DLIR software for radiation dose optimization in pediatric radiology and to assess their methodological quality.

## 2. Materials and Methods

This systematic review was conducted in accordance with the PICO framework (population: pediatric patients undergoing radiological examinations; intervention: radiation dose optimization using commercial DLIR software; comparison: DLIR versus standard practice; outcomes: performance in terms of radiation dose reduction and image quality) and the Preferred Reporting Items for Systematic Reviews and Meta-Analyses (PRISMA) guidelines (PRISMA 2020 Checklist available in Supplementary Table S1) [2,3,29,30,31,32]. The review process comprised a literature search, paper selection, data extraction, and data synthesis [2,3,4,29,30,31]. The review was registered with the Open Science Framework (Registration DOI: 10.17605/OSF.IO/3JDQS) [31].

### 2.1. Literature Search

The literature search was conducted on 28 April 2026 using seven electronic databases: *Institute of Electrical and Electronics Engineers (IEEE) Xplore*, *PubMed*, *ScienceDirect*, *Scopus*, *SpringerLink*, *Web of Science*, and *Wiley Online Library*. The search aimed to identify original clinical studies evaluating commercial DLIR software for radiation dose optimization in pediatric radiology [29,30,31]. The search statement used, (“Artificial Intelligence” OR “Machine Learning” OR “Deep Learning”) AND (“Dose Optimization” OR “Dose Reduction”) AND (“Pediatric” OR “Children”) AND (“Radiology” OR “Medical Imaging”), was informed by a previous systematic review on the performance of in-house and commercial DLIR software for radiation dose optimization in pediatric radiology [4]. No publication year restrictions were applied [29,30,31].

### 2.2. Article Selection

Article selection was performed by a single reviewer with more than 20 years of experience in conducting literature reviews. Table 1 summarizes the criteria for paper selection [2,3,4,29,30].

**Figure 1:**
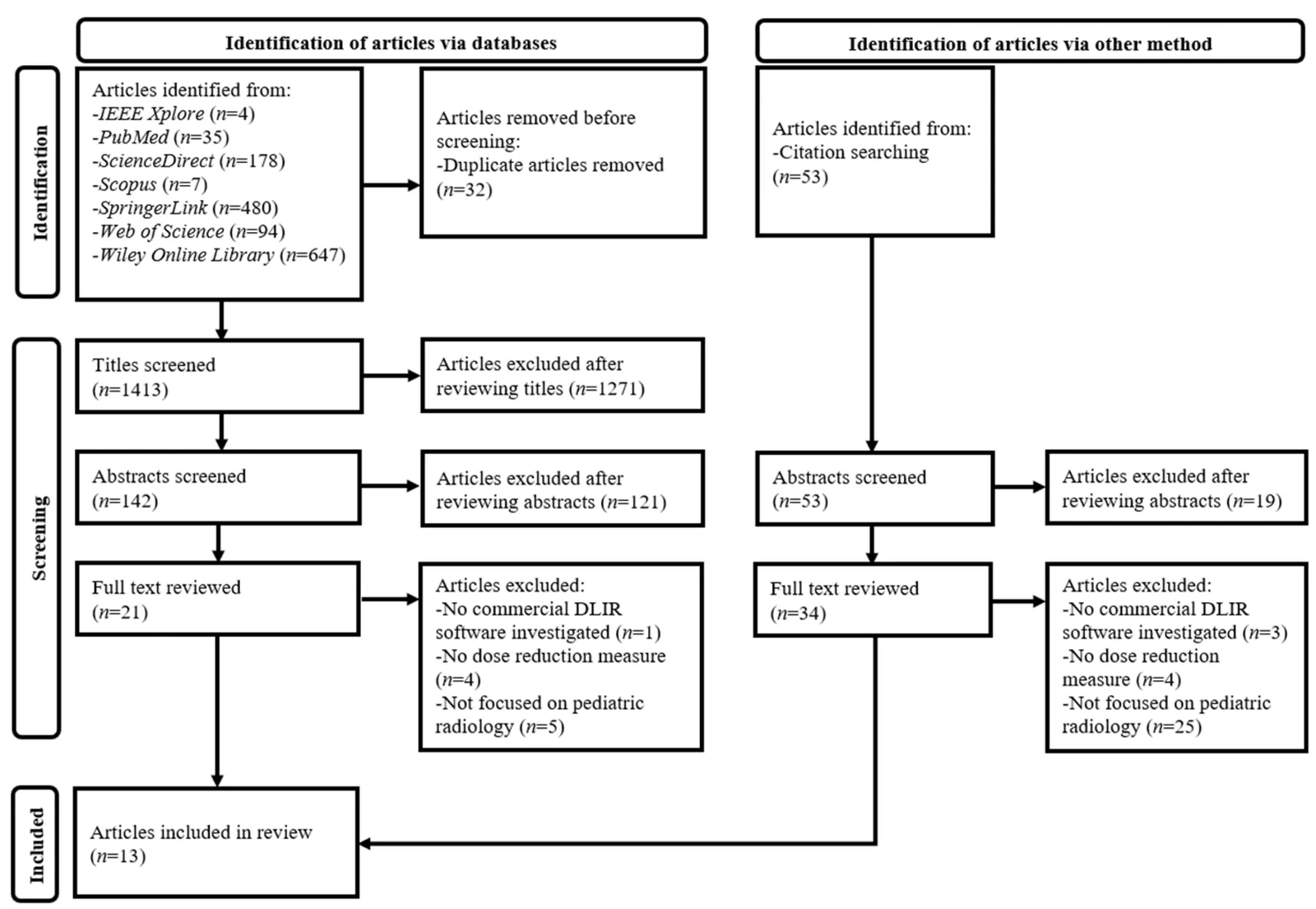
Preferred Reporting Items for Systematic Reviews and Meta-Analyses flow diagram for the systematic review of the performance of commercial deep learning-based image reconstruction (DLIR) software for radiation dose optimization in pediatric radiology. IEEE, Institute of Electrical and Electronics Engineers.

The selection criteria were established in line with the purpose of this review, which was to evaluate the performance of commercial DLIR software for radiation dose optimization in pediatric radiology, as reported in the included original clinical studies, and to assess the methodological quality of these studies [2,3,4,29,30]. Conference proceedings were excluded because of their generally lower academic rigor, making them less suitable for methodological appraisal in a systematic review [29,30,33,34]. Figure 1 illustrates the study selection process, including deduplication, screening of titles and abstracts, and full text assessment based on the predefined criteria. Articles were retained unless a clear exclusion decision could be made. Additional relevant studies were identified through backward reference searching, i.e., screening the reference lists of the included studies [2,3,4,29,30].

### 2.3. Data Extraction and Synthesis

A data extraction form was developed based on three previous systematic reviews on the performance of in-house and commercial DLIR software for radiation dose optimization in pediatric radiology [4], as well as on commercial DL-based auto-segmentation (DLAS) software for breast and prostate cancer radiation therapy planning [29,30]. Data extracted from each included paper comprised: author and country; year of publication; software product name (e.g., Canon Medical Systems’ AiCE); model architecture (e.g., convolutional neural network (CNN)); study design (e.g., prospective); multi-center involvement (yes/no); study population (e.g., patients aged 0.0–3.0 years); testing dataset source (e.g., private dataset from one Italian center), dataset size (number of patients/studies), and sample size calculation (yes/no); external testing (i.e., evaluation of the model using a dataset obtained from a source different from that used for model development; yes/no); application modality (e.g., CT) and examination type (e.g., cardiac CT angiography); reference standard (e.g., statistical-based iterative reconstruction (SBIR)); dose (e.g., effective dose (ED)) reduction performance relative to the reference standard; and changes in objective image quality (e.g., noise reduction) and subjective image quality relative to the reference standard [4,29,30].

For articles that evaluated DLIR software performance using both phantom and patient data, and/or both adult and pediatric patient data, and/or multiple reference standards, only the best mean testing results (or median values if the mean was not available) related to pediatric patients were extracted. In addition, when studies reported multiple subjective evaluation outcomes, only overall diagnostic accuracy/confidence and image quality metrics were extracted, as these are more clinically relevant and facilitate performance comparison. The data synthesis strategies employed in this review included [4,29,30]:

1. Calculating average values for individual results when overall figures for objective and subjective image quality evaluations were not reported;
2. Calculating average values from different demographic subgroups to represent overall results when such overall values were not available (while also reporting subgroup-specific results where applicable);
3. Calculating percentages of dose reduction and/or image quality change when these values were not explicitly reported using the equation: [(reference standard value—DLIR value)/reference standard value] X 100%.

Given that the included studies employed a range of evaluation approaches, resulting in substantial heterogeneity, meta-analysis was considered to have limited value and was therefore not performed [2,3,29,30,31,35]. The revised Checklist for Artificial Intelligence in Medical Imaging (CLAIM) 2024 was used to assess the quality of the included papers through evaluation of CLAIM adherence [36,37,38,39].

## 3. Results

Thirteen papers met the selection criteria and were included in this systematic review. Although studies evaluating commercial DLIR software for PET and X-ray imaging were identified during the literature search process, they did not meet the selection criteria and were therefore excluded during screening (Table 1). For example, Theruvath et al.’s study of Subtle Medical’s SubtlePET included participants aged 6–30 years and did not report findings separately for pediatric patients [20]. In addition, Fujikawa et al.’s study of Canon Medical Systems’ INR for X-ray imaging did not include a radiation dose reduction assessment [21]. Consequently, all included studies evaluated CNN-based DLIR software for CT and used datasets collected from a single private center for external testing [40,41,42,43,44,45,46,47,48,49,50,51,52]. Collectively, these studies included patients across all pediatric age groups (0–1, >1–2, 3–5, 6–11, and 12–18 years), with each study covering more than one age group [40,41,42,43,44,45,46,47,48,49,50,51,52,53]. They evaluated five software products, including Canon Medical Systems’ AiCE [40,41,42], ClariPI’s ClariCT.AI [43], GE Healthcare’s TrueFidelity [44,45,46,47,48,49,50], Philips Healthcare’s Precise Image [51], and United Imaging Healthcare’s Artificial Intelligence Iterative Reconstruction (AIIR) (Shanghai, People’s Republic of China) [52].

Table 2 summarizes the characteristics of the included articles [40,41,42,43,44,45,46,47,48,49,50,51,52]. Approximately 40% of the papers (5/13) were published from 2025 onward [42,49,50,51,52], while the remainder were published between 2021 and 2022 [40,41,43,44,45,46,47,48]. Approximately half of the studies (6/13) used pediatric datasets from China [44,45,46,48,49,52], and overall, around 70% (9/13) of the testing datasets involved pediatric patient populations from Asia [41,42,43,44,45,46,48,49,52]. Just over half of the studies (7/13) were retrospective [40,41,42,43,46,47,50], while the remainder collected data prospectively for DLIR evaluation [44,45,48,49,51,52]. In all but one of these studies, retrospectively acquired data were used as the reference standard for comparison, resulting in a prospective–retrospective study design [44,45,49,51,52].

Among the included studies, all common pediatric CT examinations were covered, including head, chest, and abdominal CT [40,41,42,43,44,45,46,47,48,49,50,51,52,54]. Unenhanced chest CT was the most common application (5/13, 38.5%) for DLIR evaluation [47,48,50,51,52]. All but one study used iterative reconstruction (IR) as the reference standard [40,41,42,43,44,45,46,47,48,49,51,52], with 11 studies (84.6%) specifically using hybrid iterative reconstruction (HIR) to provide reference values for radiation dose and image quality comparison [41,42,43,44,45,46,47,48,49,51,52]. The only exception relied on international diagnostic reference levels (DRLs) and a pediatric radiologist’s judgement for performance evaluation [50].

**Figure 2:**
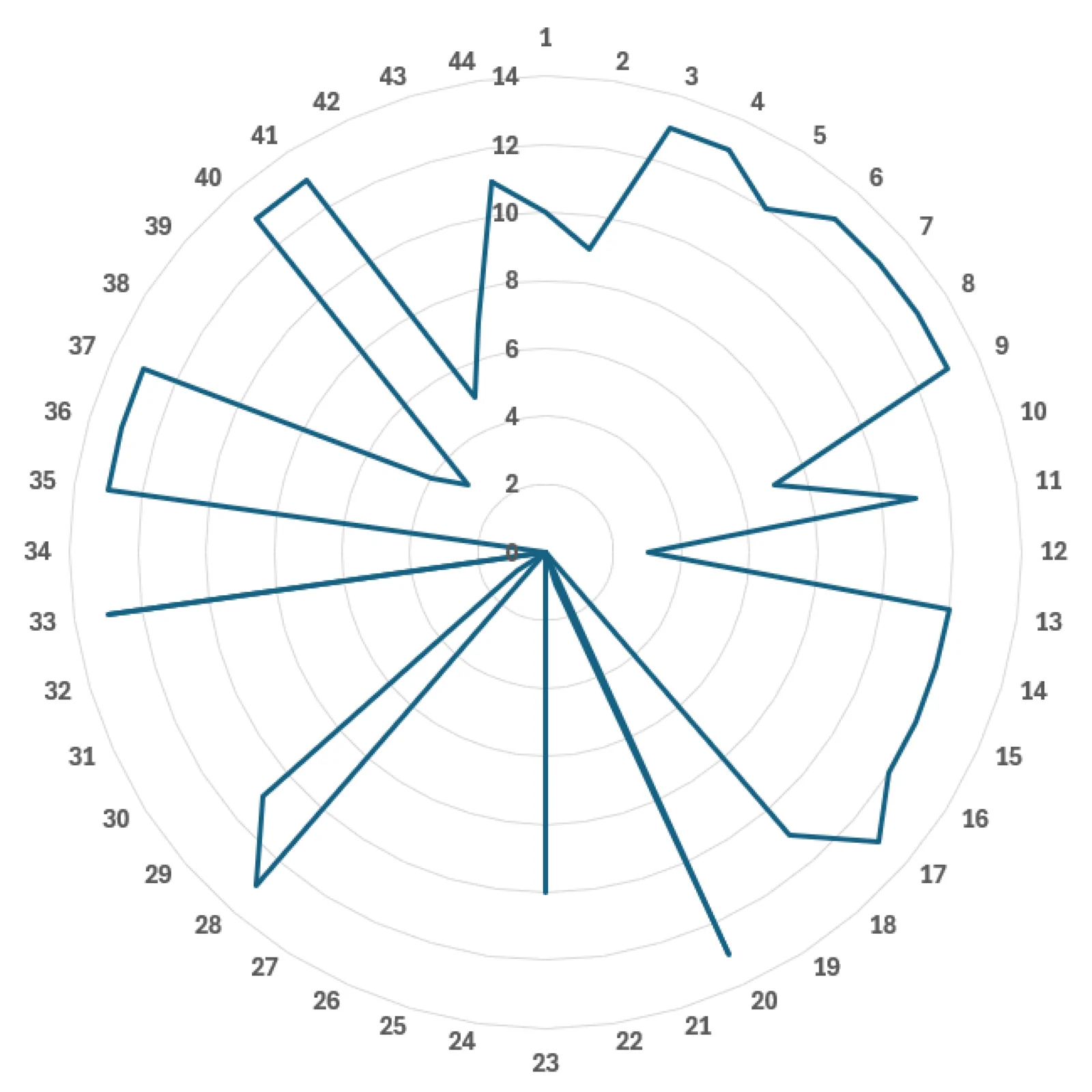
Number of included articles meeting each criterion of the revised Checklist for Artificial Intelligence in Medical Imaging (CLAIM) 2024. Radial axis represents the number of studies; angular axis represents the CLAIM 2024 criteria: 1. artificial intelligence methodology specified in title; 2. abstract summary adequacy; 3. scientific/clinical background; 4. study aims/objectives/hypotheses; 5. prospective/retrospective design; 6. study goal; 7. data sources; 8. inclusion/exclusion criteria; 9. data preprocessing; 10. selection of data subsets; 11. de-identification methods; 12. missing data handling; 13. image acquisition protocol; 14. reference standard definition; 15. reference standard rationale; 16. source of reference standard annotations; 17. test set annotation; 18. inter/intrarater variability; 19. data partition assignment; 20. disjoint partition level; 21. intended sample size; 22. detailed model description; 23. software libraries/frameworks/packages; 24. model parameter initialization; 25. training approach details; 26. final model selection; 27. ensembling technique; 28. model performance metrics; 29. statistical significance/uncertainty measures; 30. robustness/sensitivity analysis; 31. explainability/interpretability methods; 32. internal data evaluation; 33. external data testing; 34. clinical trial registration; 35. included/excluded patient/examination numbers; 36. demographic/clinical characteristics of dataset; 37. performance metrics with uncertainty; 38. diagnostic performance estimates and precision; 39. failure analysis; 40. study limitations; 41. practice implications/clinical role; 42. protocol/technical details; 43. software/model/data availability; and 44. funding/support and funder role.

The range of CLAIM adherence scores of the included papers was 56.0–74.0% (mean: 64.2%; median: 62.8%). Figure 2 illustrates the number of papers meeting each criterion of the revised CLAIM 2024. The main methodological weaknesses identified across these studies were the limited reporting of DLIR model architectures and the lack of detailed information on training processes, given the proprietary nature of the commercial software [40,41,42,43,44,45,46,47,48,49,50,51,52]. Only one study reported the calculation of the sample size required to detect a significant change in radiation dose and indicated that at least 71 patients were needed [51]. However, only approximately one-quarter of the included studies (3/13), including that study, used datasets with at least 71 patients for DLIR software evaluation [50,51,52]. The range of testing dataset sizes was 19–191 patients [40,41,42,43,44,45,46,47,48,49,50,51,52].

Table 3 shows the radiation dose reduction performance of commercial DLIR software covered by the included studies. The most commonly reported dose metric (10/13, 76.9%) was volume CT dose index (CTDI_vol_) [40,43,44,45,47,48,49,50,51,52], followed by dose-length product (DLP), which was assessed in eight studies (61.5%) [43,44,45,47,49,50,51,52].

Regardless of the radiation dose metric used, the mean/median dose reduction achieved with DLIR across the included studies ranged from 11.2% (GE Healthcare’s TrueFidelity [44]) to 97.9% (GE Healthcare’s TrueFidelity [50]). For Canon Medical Systems’ AiCE, the mean dose reduction ranged from 50.0% to 59.5% [40,41,42]. Among software evaluated in single studies, ClariPI’s ClariCT.AI demonstrated the lowest mean dose reduction (19.6% in terms of CTDI_vol_) [43], whereas United Imaging Healthcare’s AIIR achieved the highest performance (90.5% reduction in DLP) [52]. Philips Healthcare’s Precise Image showed an intermediate performance, with median dose reductions of 61.9% (CTDI_vol_), 70.1% (DLP), and 72.1% (ED) [51]. Overall, more than three-quarters of the included papers (10/13) reported dose reductions of at least 50% [40,41,42,46,47,48,49,50,51,52]. For studies with *p*-values available, all dose reductions were statistically significant (*p* < 0.0001–0.01) [41,42,43,44,45,47,49,51].

Table 4 illustrates the objective and subjective image quality evaluation results of the DLIR software. The most commonly reported evaluation metrics (10/13, 76.9%) were the standard deviation of CT number for objective noise measurement [41,42,43,44,45,46,47,49,51,52] and subjective image quality assessment by radiologists [40,41,43,44,45,46,47,48,49,52]. The second most commonly reported objective and subjective evaluation metrics were contrast-to-noise ratio (CNR), assessed in nine studies (69.2%) [41,42,43,44,45,46,47,48,49], and diagnostic confidence/quality, reported in five papers (38.5%) [42,43,45,50,51], respectively. Regarding image quality, ten studies [41,42,43,44,45,46,47,49,51,52] reported changes in mean/median objective image noise ranging from a 0.4% increase (GE Healthcare’s TrueFidelity [45]) to a 62.2% reduction (Philips Healthcare’s Precise Image [51]). Mean CNR changes, reported by nine studies [41,42,43,44,45,46,47,48,49], ranged from a 31.3% decrease (GE Healthcare’s TrueFidelity [49]) to a 75.8% increase (GE Healthcare’s TrueFidelity [47]). For subjective image quality, ten studies [40,41,43,44,45,46,47,48,49,52] reported changes ranging from an 8.4% decrease (GE Healthcare’s TrueFidelity [49]) to a 45.5% increase (GE Healthcare’s TrueFidelity [48]). Diagnostic confidence/quality, reported in five studies [42,43,45,50,51], ranged from a 7.6% decrease (ClariPI’s ClariCT.AI [43]) to a 25.0% increase (Canon Medical Systems’ AiCE [42]).

Nevertheless, with the exception of Li et al.’s [49] study, which used DLIR to maintain image quality and diagnostic accuracy despite reductions in kV, mA, contrast medium volume, and contrast injection flow rate, all other studies (6/7, 85.7%) that showed increased image noise and/or decreased CNR, subjective image quality, or diagnostic confidence/quality found that these differences were not statistically significant compared with the reference standard (*p* = 0.092–0.94) [43,45,46,47,48,52]. Li et al. [49] reported that DLIR resulted in decreases in SNR and CNR of 10.4% (*p* = 0.092) and 31.3% (*p* < 0.001), respectively, as well as a decrease in overall image quality of 8.4% (*p* < 0.001) compared with the reference standard. However, the DLIR images remained diagnostically acceptable, with 100% sensitivity and specificity reported.

## 4. Discussion

This is the first systematic review of the performance of commercial DLIR software for radiation dose optimization in pediatric radiology, covering 13 studies and five CT DLIR software products (Canon Medical Systems’ AiCE, ClariPI’s ClariCT.AI, GE Healthcare’s TrueFidelity, Philips Healthcare’s Precise Image, and United Imaging Healthcare’s AIIR), with the evaluation of their radiation dose reduction capabilities and impacts on image quality [40,41,42,43,44,45,46,47,48,49,50,51,52]. In 2021, Nagayama et al. [5] published a narrative review on DLIR in pediatric CT and reported that DLIR could reduce CT radiation dose by 30–80% compared with standard practice using HIR, based on two adult phantom studies involving GE Healthcare’s TrueFidelity [55,56] and two adult clinical studies evaluating Canon Medical Systems’ AiCE [57,58]. In 2022, Ng [4] published a systematic review on DLIR in pediatric radiology that included 11 studies (10 clinical studies and one phantom study; nine using commercial products and two using in-house models) that evaluated the dose reduction performance of DLIR and reported that DLIR could achieve 11–95% dose reduction while maintaining acceptable image quality. It should be noted that the adult phantom studies cited in Nagayama et al.’s review [5], as well as the two studies using in-house DLIR models (including one phantom study) covered in Ng’s systematic review [4], were not eligible for inclusion in this review because only original clinical studies evaluating commercial DLIR software were considered. These studies are discussed solely to facilitate a comparison with previous review findings and were not included in the evidence synthesis of this review.

This systematic review included 13 clinical studies and evaluated two CT DLIR products that were not covered in the previous reviews, namely, Philips Healthcare’s Precise Image and United Imaging Healthcare’s AIIR [4,5]. Furthermore, it demonstrated that CT DLIR could achieve dose reductions ranging from 11.2% to 97.9% without compromising image quality, and in some situations, even exceeded that of the reference standard. Excluding two studies that reported substantially lower dose reductions [43,44], the clinically achievable dose reduction range in pediatric CT was 36.0–97.9% [40,41,42,45,46,47,48,49,50,51,52]. Therefore, the findings of this review are consistent with those of previous reviews but are supported by a broader and more clinically relevant evidence base, thereby extending current knowledge on the performance of commercial DLIR software in pediatric CT [4,5,40,41,42,43,44,45,46,47,48,49,50,51,52].

As noted in Table 3, one of the main factors contributing to the wide variation in radiation dose reduction performance appears to be differences among commercial DLIR software products. For example, the mean dose reduction achieved by Canon Medical Systems’ AiCE ranged from 50.0% to 59.5% [40,41,42], whereas ClariPI’s ClariCT.AI demonstrated a considerably lower mean dose reduction (19.6%) [43] and United Imaging Healthcare’s AIIR achieved a substantially higher reported dose reduction (90.5%) [52]. Philips Healthcare’s Precise Image demonstrated an intermediate performance, with median dose reductions ranging from 61.9% to 72.1% [51]. For the same DLIR software product, differences in age groups, examination types, reference standards, and dose metrics generally appeared to have a smaller impact on the reported dose reduction performance than differences between software products, with variations typically around 10% or less [40,41,42,43,44,45,46,47,48,49,50,51,52].

However, substantial variation was observed for GE Healthcare’s TrueFidelity, with reported dose reductions ranging from 11.2% to 97.9%. This wide range was mainly attributable to differences in the CT scanning protocols used across the included studies [44,45,46,47,48,49,50]. For example, Sun et al. [44] reported the lowest dose reduction achieved with GE Healthcare’s TrueFidelity (CTDI_vol_: 13.3% and DLP: 11.2%) for chest CT angiography, but also reported a 39.8% improvement in image quality. This finding suggests the possibility that further radiation dose reduction may be achievable through the use of lower-dose scanning protocols while maintaining image quality comparable to that of the reference standard, HIR; however, further clinical validation is required.

Regarding the metrics used to assess DLIR performance, CTDI_vol_, DLP, objective image noise and CNR evaluations, and subjective image quality and diagnostic confidence/quality assessments were the most commonly and second most commonly used metrics, respectively [40,41,42,43,44,45,46,47,48,49,50,51,52]. CTDI_vol_ and DLP are readily available from CT dose reports [52]. However, size-specific dose estimate (SSDE) and ED can provide patient-specific dose information and facilitate dose comparisons across different imaging modalities, respectively, although additional calculations are required [40,41,42,43,45,47,50,51,52]. Objective image noise and CNR enable the direct evaluation of DLIR performance because DLIR is fundamentally an image denoising application, and image noise influences image contrast, which may subsequently affect diagnostic confidence [41,42,43,44,45,46,47,48,49,51,52]. However, the assessment of diagnostic confidence/quality/accuracy by pediatric radiologists is arguably more important because these measures reflect the real-world clinical impact of DLIR and have been recommended by the ACR, AOSPR, ESPR, SLARP, SPIN, and SPR [26]. Nonetheless, only about half of the studies included in this review evaluated diagnostic confidence/quality/accuracy [42,43,45,49,50,51].

Based on the currently available evidence, clinicians should consider the consistency of radiation dose reduction performance, effects on diagnostic confidence/quality/accuracy, the intended examination type, and CT platform compatibility when selecting a commercial DLIR product for clinical use [26,40,41,42,43,44,45,46,47,48,49,50,51,52]. Among the included studies [40,41,42,43,44,45,46,47,48,49,50,51,52], Canon Medical Systems’ AiCE demonstrated the most consistent reported performance for abdominal contrast-enhanced CT, achieving radiation dose reductions of 50.0–59.5% together with 12.9–25.0% improvements in image quality/diagnostic confidence [40,41,42]. In contrast, the reported performance of GE Healthcare’s TrueFidelity was more variable, even for similar anatomical regions and examination types [44,45,46,47,48,49,50].

According to the joint statement on the implementation of AI in pediatric radiology published in 2025 by the ACR, ESPR, SPR, SLARP, AOSPR, and SPIN, clinical centers should monitor the diagnostic performance of DLIR in real-world settings across diverse pediatric populations [26]. This recommendation may partly explain why approximately 40% (5/13) of the included studies were published from 2025 onward [42,49,50,51,52]. Furthermore, four of these five studies (80.0%) evaluated the impact of DLIR on diagnostic confidence/quality/accuracy [42,49,50,51]. In addition, just over half of the studies included in this review were retrospective [40,41,42,43,46,47,50] compared with approximately two-thirds of the clinical studies included in Ng’s 2022 systematic review [4]. This reduction in the proportion of retrospective studies is consistent with the recommendation outlined in CLAIM 2024 [39]. Furthermore, Bianco et al. [51], published in 2026, conducted a sample size calculation to ensure the generalizability of the evaluation findings [39]. All three studies that met the minimum sample size of 71 patients proposed by Bianco et al. [51] for detecting clinically meaningful changes in radiation dose were published from 2025 onward [50,51,52].

However, broader coverage of pediatric populations and examination types remains an important area for improvement. No included study covered the entire pediatric age range, and only CT-based DLIR studies were identified in this review (Table 2) [40,41,42,43,44,45,46,47,48,49,50,51,52]. Furthermore, approximately 70% (9/13) of the testing datasets used in the included studies involved Asian pediatric patient populations [41,42,43,44,45,46,48,49,52]. Therefore, future clinical studies should evaluate a broader range of commercial DLIR software, including AlgoMedica’s PixelShine [12], Subtle Medical’s SubtlePET [20], Canon Medical Systems’ INR [21,22], and Samsung Electronics Co., Ltd.’s SimGrid [23]. These studies should ideally cover the full pediatric age range, include prospectively collected datasets from underrepresented regions such as Africa, South America, and Oceania, recruit at least 71 patients, and assess radiation dose reduction using ED alongside diagnostic confidence/accuracy outcomes.

Despite these limitations, the CLAIM adherence scores of the included studies ranged from 56.0% to 74.0% (mean: 64.2%; median: 62.8%), which were numerically higher than those reported in previous systematic reviews by the author of commercial DLAS software for breast (range: 44.0–65.0%; mean: 53.9%; median: 53.0%) and prostate cancer radiation therapy planning (range: 40.0–72.0%; mean: 52.1%; median: 53.0%). However, direct comparison should be interpreted cautiously given the differences in clinical applications, evidence bases, and study characteristics between the reviews [29,30].

This systematic review had several major limitations. First, article selection, data extraction and synthesis, and CLAIM adherence assessments were conducted by a single reviewer. Although the reviewer had more than 20 years of experience conducting literature reviews and used predefined article selection criteria [2,3,4,29,30], the PRISMA guidelines [32], a structured data extraction form [4,29,30], and the CLAIM [36,39], the absence of independent verification may have increased the risk of extraction, synthesis, and appraisal bias.

Second, CLAIM was originally developed to improve the transparency and reproducibility of AI studies in medical imaging rather than to function as a formal risk-of-bias assessment tool [39]. Nonetheless, CLAIM has subsequently been used to evaluate the methodological and reporting quality of AI studies in medical imaging. Previous systematic reviews have employed CLAIM as a framework for assessing the methodological quality of AI studies [59,60]. Furthermore, recent umbrella-review evidence has demonstrated that CLAIM adherence is associated with journal impact-factor quartile, with studies published in higher-quartile journals generally demonstrating higher levels of CLAIM adherence. As higher-quartile journals typically apply more rigorous standards during the peer-review process, these findings suggest that CLAIM adherence may provide useful insights into the overall methodological rigor and reporting completeness of AI studies. However, CLAIM primarily evaluates methodological and reporting quality rather than directly assessing risk of bias. Therefore, higher CLAIM scores should not automatically be interpreted as indicating a lower risk of bias [61].

In addition, only peer-reviewed original clinical studies published in English that evaluated the performance of commercial DLIR software and reported radiation dose reduction outcomes were included. These selection criteria may have limited the number of eligible papers. However, the number of clinical studies included in this review (*n* = 13) was greater than that included in Ng’s systematic review on DLIR in pediatric radiology published in 2022 (*n* = 10) [4]. Furthermore, this review covered five commercial CT DLIR software products compared with three in Ng’s systematic review [4] and two in Nagayama et al.’s narrative review published in 2021 [5]. Taken together, these findings suggest that the search strategy achieved reasonably comprehensive coverage of the currently available literature. Nevertheless, future reviews may benefit from incorporating commercial product names, additional search terms related to image denoising and iterative reconstruction, and forward citation searching.

## 5. Conclusions

This systematic review evaluated five commercial CT DLIR software products and demonstrated that clinically achievable radiation dose reductions in pediatric CT range from 11.2% to 97.9% without compromising image quality, and in some cases, even exceeded that of the reference standard, suggesting further dose reduction potential. Excluding two studies that reported substantially lower dose reductions, the clinically achievable dose reduction range was 36.0–97.9%. Furthermore, more than three-quarters of the included studies reported dose reductions of at least 50% while maintaining image quality. Improvements in study methodology were evident among papers published from 2025 onward. However, further clinical studies are needed to evaluate a broader range of commercial DLIR software, including applications in PET and X-ray imaging. Future studies should ideally cover the full pediatric age range, include prospectively collected datasets from underrepresented regions such as Africa, South America, and Oceania, recruit at least 71 patients, and assess radiation dose reduction using ED alongside diagnostic confidence/quality/accuracy outcomes.

## Figures and Tables

**Table 1:** Paper selection criteria.

Inclusion Criteria	Exclusion Criteria
Peer-reviewed, original clinical study Evaluation of performance of commercial deep learning-based image reconstruction (DLIR) software for radiation dose optimization in pediatric radiology Written in English	Commentary Conference proceeding including abstract Editorial Gray literature No dose reduction measure Non-peer-reviewed paper (e.g., those published on arXiv platform) Opinion Perspective Phantom study Review Study solely evaluating in-house DLIR model Study without findings specific to pediatric patients

**Table 2:** Characteristics of studies on commercial deep learning-based image reconstruction software for radiation dose optimization in pediatric radiology.

Author, Year, and Country	Study Design	Patient/Population	Testing Dataset		Sample Size Calculation	Examination Type	Reference Standard	CLAIM Adherence Score (%)
			Source ^1^	Size: Number of Patients				
*Canon Medical Systems’ Advanced Intelligent Clear-IQ Engine*								
Brady et al. (2021), USA [40]	R	3.0–19.0 y (mean: 11.0 ± 5.0 y)	USA	19	No	Chest-abdomen-pelvis CECT	FBP, SBIR, and MBIR	65
Nagayama et al. (2022), Japan [41]	R	0.0–6.6 y (mean: 2.2 ± 2.1 y)	Japan	34	No	Abdomen CECT	HIR	74
Harai et al. (2026), Japan [42]	R	0.0–6.0 y (mean: 2.1 ± 2.0 y)	Japan	41	No	Abdomen CECT	HIR	70
*ClariPI’s ClariCT.AI*								
Lee et al. (2021), Republic of Korea [43]	R	2.0–19.0 y (mean: 10.1 y)	South Korea	29	No	Abdomen DECT	SECT with HIR	65
*General Electric Healthcare’s TrueFidelity*								
Sun et al. (2021), People’s Republic of China and USA [44]	P–R	0.3–13.0 y (mean: 5.9 ± 4.2 y)	China	46	No	Chest CTA	HIR	60
Sun et al. (2021), People’s Republic of China and USA [45]	P–R	5.0–14.0 y (mean: 9.3 ± 3.1 y)	China	27	No	Cardiac CTA	HIR	60
Sun et al. (2021), People’s Republic of China and USA [46]	R	0.1–14.0 y (median: 2.0 y)	China	50	No	Head UECT	HIR	65
Tschauner et al. (2021), Austria and Switzerland [47]	R	2.0–18.2 y (mean: 11.1 ± 5.0 y)	Switzerland	23	No	Chest UECT	HIR	63
Zhang et al. (2022), People’s Republic of China [48]	P	4.0–6.0 y (mean: 5.4 ± 1.2 y)	China	20 (10 abdomen and 10 chest)	No	Abdomen and chest UECT	HIR	63
Li et al. (2025), People’s Republic of China [49]	P–R	Mean: 1.5 ± 1.4 y	China	53	No	Chest CTA	HIR	63
Sturm et al. (2026), Austria, Germany, Switzerland, and USA [50]	R	0.3–17.9 y (mean: 11.2 ± 4.1 y)	Switzerland	106 (277 studies)	No	Chest UECT	International DRLs for dose and 1 pediatric radiologist for image quality	56
*Philips Healthcare’s Precise Image*								
Bianco et al. (2026), Italy [51]	P–R	0.0–16.0 y (median: 10.0 y; IQR: 5.0–13.0 y)	Italy	71	Yes	Chest UECT	HIR	67
*United Imaging Healthcare’s Artificial Intelligence Iterative Reconstruction*								
Zhang et al. (2025), People’s Republic of China [52]	P–R	0.0–3.0 y (mean: 0.6 ± 0.6 y)	China	191	No	Chest UECT	Cardiac CTA with HIR	63

^1^ All datasets were sourced from a single private center; therefore, only the country of origin is reported. CECT, contrast-enhanced computed tomography; CLAIM, Checklist for Artificial Intelligence in Medical Imaging; CTA, computed tomography angiography; DECT, dual-energy computed tomography; DRLs, diagnostic reference levels; FBP, filtered back projection; HIR, hybrid iterative reconstruction; IQR, interquartile range; MBIR, model-based iterative reconstruction; P, prospective; R, retrospective; SBIR, statistical-based iterative reconstruction; SECT, single-energy polychromatic computed tomography; UECT, unenhanced computed tomography; y, year.

**Table 3:** Radiation dose reduction performance of commercial deep learning-based image reconstruction software for radiation dose optimization in pediatric radiology.

Author, Year, and Country	Dose Reduction Performance ^1^
*Canon Medical Systems’ Advanced Intelligent Clear-IQ Engine*	
Brady et al. (2021), USA [40]	CTDI_vol_ and ED (versus SBIR): 52.4% and 50.0% ^2^
Nagayama et al. (2022), Japan [41]	SSDE: 53.7% (*p* < 0.001)
Harai et al. (2026), Japan [42]	SSDE: 59.5% (*p* < 0.001)
*ClariPI’s ClariCT.AI*	
Lee et al. (2021), Republic of Korea [43]	CTDI_vol_, DLP, and ED: 19.6% (*p* < 0.0001), 21.7% (*p* = 0.0001), and 21.7% (*p* < 0.0001)
*General Electric Healthcare’s TrueFidelity*	
Sun et al. (2021), People’s Republic of China and USA [44]	CTDI_vol_ and DLP: 13.3% (*p* = 0.01) and 11.2% (*p* = 0.01)
Sun et al. (2021), People’s Republic of China and USA [45]	CTDI_vol_, DLP, and ED: 37.5% (*p* = 0.01), 42.8% (*p* = 0.01), and 36.0% (*p* = 0.01)
Sun et al. (2021), People’s Republic of China and USA [46]	85.0% ^2,3^
Tschauner et al. (2021), Austria and Switzerland [47]	ED: overall (79.6% (*p* < 0.001)), 0–5 y (77.8% ^2^), 6–10 y (78.7% ^2^), 11–15 y (77.9% ^2^), and 16–18 y (82.2% ^2^); overall CTDI_vol_, DLP and SSDE: 72.9% (*p* < 0.001), 79.5% (*p* < 0.001), and 73.0% (*p* < 0.001)
Zhang et al. (2022), People’s Republic of China [48]	CTDI_vol_: abdomen (57.0%) and chest (53.9%) ^2^
Li et al. (2025), People’s Republic of China [49]	CTDI_vol_ and DLP: 49.2% (*p* < 0.001) and 51.5% (*p* < 0.001)
Sturm et al. (2026), Austria, Germany, Switzerland, and USA [50]	Median CTDI_vol_, DLP, and SSDE: 0–5 y (96.4%, 97.6%, and 94.6%); 5–10 y (95.2%, 96.8%, and 93.1%); 10–15 y (96.2%, 97.3%, and 94.1%); >15 y (97.3%, 97.9%, and 95.5%) ^2^
*Philips Healthcare’s Precise Image*	
Bianco et al. (2026), Italy [51]	Median CTDI_vol_, DLP and ED: overall (61.9% (*p* < 0.001), 70.1% (*p* < 0.001), and 72.1%; (*p* < 0.001)); 0–5 y (67.0% (*p* < 0.001), 67.7% (*p* < 0.001), and 72.9% (*p* < 0.001)); 6–10 y (67.8% (*p* < 0.001), 68.7% (*p* < 0.001), and 70.6% (*p* < 0.001)); 11–16 y (70.0% (*p* < 0.001), 69.5% (*p* < 0.001), and 68.1% (*p* < 0.001))
*United Imaging Healthcare’s Artificial Intelligence Iterative Reconstruction*	
Zhang et al. (2025), People’s Republic of China [52]	CTDI_vol_, DLP and ED: 89.7%, 90.5%, and 90.4% ^2^

^1^ Unless otherwise specified, all values are reported as mean values. ^2^ *p*-values were not available. ^3^ Radiation dose metric was not specified. CTDI_vol_, volume computed tomography dose index; DLP, dose-length product; ED, effective dose; SBIR, statistical-based iterative reconstruction; SSDE, size-specific dose estimate; y, year.

**Table 4:** Objective and subjective image quality evaluation results of commercial deep learning-based image reconstruction software for radiation dose optimization in pediatric radiology.

Author, Year, and Country	Image Quality ^1^	
	Objective	Subjective
*Canon Medical Systems’ Advanced Intelligent Clear-IQ Engine*		
Brady et al. (2021), USA [40]	Object detectability increase (vs FBP, SBIR, and MBIR): 51.0%, 18.0%, and 11.0% ^2^	Image quality increases (vs FBP, SBIR, and MBIR): 52.2% (*p* < 0.001), 12.9% (*p* < 0.001), and 12.9% (*p* < 0.001)
Nagayama et al. (2022), Japan [41]	Noise reduction: 6.8% (*p* < 0.001); SNR/CNR increase: 6.5% (*p* < 0.05)/15.9% (*p* < 0.05)	Image quality increase: 22.6% (*p* < 0.001)
Harai et al. (2026), Japan [42]	Noise reduction: 9.4% (*p* = 0.017); CNR (aorta/liver/portal vein) and ERS increases: 6.5% (*p* = 0.340)/1.4% (*p* = 0.827)/8.2% (*p* = 0.184) and 20.6% (*p* = 0.011)	Diagnostic confidence increase: 25.0% (*p* < 0.001)
*ClariPI’s ClariCT.AI*		
Lee et al. (2021), Republic of Korea [43]	Noise reduction: 1.8% (*p* = 0.024); SNR/CNR change: aorta (5.0% (*p* = 0.177)/1.6% (*p* = 0.758)), pancreas (4.0% (*p* = 0.206)/−7.7% (*p* = 0.283)), and liver (9.6% (*p* = 0.006)/6.7% (*p* = 0.300))	Diagnostic and image quality changes: −7.6% (*p* = 0.096) and −4.9% (*p* = 0.111)
*General Electric Healthcare’s TrueFidelity*		
Sun et al. (2021), People’s Republic of China and USA [44]	Noise reduction (aorta/muscle): 24.7% (*p* < 0.001)/18.8% (*p* < 0.001); SNR/CNR increase: 79.8% (*p* < 0.001)/62.3% (*p* < 0.001)	Image quality increase: 39.8% ^2^
Sun et al. (2021), People’s Republic of China and USA [45]	Noise change (aorta/muscle): −12.0% (*p* < 0.001)/0.4% (*p* = 0.94); SNR/CNR change: −0.6% (*p* = 0.92)/−8.6% (*p* = 0.19)	Diagnostic confidence and image quality increases: 0.0% (*p* = 0.84) and 6.7% (*p* = 0.13)
Sun et al. (2021), People’s Republic of China and USA [46]	Noise (GM/WM) reduction: 0.0%/1.6%; SNR (GM/WM) and CNR changes: −2.5%/1.4% and −1.7% ^2^	Image quality change: −0.3% ^2^
Tschauner et al. (2021), Austria and Switzerland [47]	Background noise reduction: 44.1% (*p* < 0.001); SNR (liver/heart) and CNR increases: 240.0% (*p* < 0.001)/96.2% (*p* < 0.001) and 75.8% (*p* < 0.001)	Image quality change: −0.6% ^2^
Zhang et al. (2022), People’s Republic of China [48]	SNR changes (abdomen-fat/kidney/liver and chest-lung/mediastinum/spine): 76.1%/33.3%/45.8% and −30.3%/48.0%/5.3%; CNR increase (abdomen-kidney/liver): 32.8%/42.3% ^2^	Image quality increases: abdomen (45.5%) and chest (6.9%) ^2^
Li et al. (2025), People’s Republic of China [49]	Noise (overall/artery/muscle) and ERD reductions: 46.8% (*p* < 0.001)/41.4% (*p* < 0.001)/8.1% (*p* = 0.026) and 1.2% (*p* = 0.356); SNR/CNR changes: −10.4% (*p* = 0.092)/−31.3% (*p* < 0.001)	Sensitivity, specificity and overall image quality changes: 0.0%,^2^ 0.0%,^2^ and −8.4% (*p* < 0.001)
Sturm et al. (2026), Austria, Germany, Switzerland, and USA [50]	Not available	Sufficient diagnostic quality
*Philips Healthcare’s Precise Image*		
Bianco et al. (2026), Italy [51]	Median noise reduction (overall/0–5 y/6–10 y/11–16 y): air (13.8% (*p* = 0.426)/27.1% (*p* > 0.05)/25.6% (*p* = 0.017)/53.7% (*p* = 0.004); aorta (9.1% (*p* = 0.529)/22.7% (*p* > 0.05)/0.0% (*p* > 0.05)/9.1% (*p* > 0.05)); lung parenchyma (22.2% (*p* = 0.017)/6.0% (*p* > 0.05)/8.6% (*p* > 0.05)/47.7% (*p* < 0.001)); muscle (20.0% (*p* < 0.001)/4.3% (*p* > 0.05)/26.7% (*p* = 0.001)/25.0% (*p* < 0.001)); trachea (24.2% (*p* < 0.001)/1.3% (*p* > 0.05)/8.3% (*p* > 0.05)/62.2% (*p* < 0.001))	Diagnostic quality increase: 2.4% ^2^
*United Imaging Healthcare’s Artificial Intelligence Iterative Reconstruction*		
Zhang et al. (2025), People’s Republic of China [52]	Noise reduction: 54.5% (*p* < 0.001)	Image quality change: −3.1% (*p* > 0.05)

^1^ Unless otherwise specified, all values are reported as mean values. ^2^ *p*-values were not available. CNR, contrast-to-noise ratio; ERD, edge rise distance; ERS, edge rise slope; FBP, filtered back projection; GM, gray matter; MBIR, model-based iterative reconstruction; SBIR, statistical-based iterative reconstruction; SNR, signal-to-noise ratio; vs, versus; WM, white matter; y, year.

## Data Availability

No new data were analyzed in this study. Data sharing is not applicable to this article.

## Supplementary Materials

The following supporting information can be downloaded at https://www.mdpi.com/article/10.3390/children13091147/s1, Table S1: PRISMA 2020 Checklist [62].

## Abbreviations

The following abbreviations are used in this manuscript:

ACR	American College of Radiology
AI	Artificial intelligence
AiCE	Advanced Intelligent Clear-IQ Engine
AIIR	Artificial Intelligence Iterative Reconstruction
ALARA	As low as reasonably achievable
AOSPR	Asian Oceanic Society of Paediatric Radiology
CECT	Contrast-enhanced computed tomography
CLAIM	Checklist for Artificial Intelligence in Medical Imaging
CNN	Convolutional neural network
CNR	Contrast-to-noise ratio
CT	Computed tomography
CTA	Computed tomography angiography
CTDI_vol_	Volume computed tomography dose index
Curtin MRI	Curtin Medical Research Institute
DECT	Dual-energy computed tomography
DL	Deep learning
DLAS	Deep learning-based auto-segmentation
DLIR	Deep learning-based image reconstruction
DLP	Dose-length product
DRLs	Diagnostic reference levels
ED	Effective dose
ERD	Edge rise distance
ERS	Edge rise slope
ESPR	European Society of Paediatric Radiology
FBP	Filtered back projection
GE	General Electric
GM	Gray matter
HIR	Hybrid iterative reconstruction
IEEE	Institute of Electrical and Electronics Engineers
INR	Intelligent noise reduction
IQR	Interquartile range
IR	Iterative reconstruction
MBIR	Model-based iterative reconstruction
P	Prospective
PET	Positron emission tomography
PRISMA	Preferred Reporting Items for Systematic Reviews and Meta-Analyses
R	Retrospective
SBIR	Statistical-based iterative reconstruction
SECT	Single-energy polychromatic computed tomography
SLARP	Sociedad Latinoamericana de Radiologia Pediatrica
SNR	Signal-to-noise ratio
SPIN	Society of Paediatric Neuroimaging
SPR	Society for Paediatric Radiology
SSDE	Size-specific dose estimate
UECT	Unenhanced computed tomography
vs	Versus
WM	White matter
y	Year
